# The role of *Mental Health Care Act* status in dignity-related complaints by psychiatric inpatients: A cross-sectional analytical study

**DOI:** 10.4102/sajpsychiatry.v27i0.1602

**Published:** 2021-05-27

**Authors:** Shonisani Raphalalani, Piet J. Becker, Manfred W. Böhmer, Christa Krüger

**Affiliations:** 1 Department of Psychiatry, Faculty of Health Sciences, University of Pretoria, Pretoria, South Africa; 2 Department of Research Office, Faculty of Health Sciences, University of Pretoria, Pretoria, South Africa

**Keywords:** legal status, patients’ rights; dignity, psychiatric admissions, dignity-related complaints, *Mental Health Care Act*

## Abstract

**Background:**

Globally interest has grown in promoting the rights of patients, especially psychiatric patients. Two core elements of patients’ rights are the rights to be treated in a dignified manner and to give feedback about services. Psychiatric patients may feel treated in an undignified manner, especially during involuntary hospital admissions.

**Aim:**

We explored the relationship between *Mental Health Care Act* 17 of 2002 (MHCA) status and dignity-related complaints.

**Setting:**

The study was conducted at a specialist state psychiatric hospital.

**Methods:**

We reviewed 120 registered complaints by psychiatric inpatients, retrieved the clinical files, and analysed 70 complaints. Fisher’s exact tests described the relationship between patients’ MHCA status and the frequency of dignity-related or other categories of complaints. Logistic regression analyses were adjusted for potential covariates.

**Results:**

Most complaints were from single, literate male patients, aged 30–39 years, with mood disorders. Most complainants were admitted involuntarily (60%). Dignity-related complaints (*n* = 41; 58%) outnumbered nondignity-related complaints (*n* = 29; 41%). The proportion of dignity-related complaints was higher in involuntary (64%) and assisted (60%) patients than in voluntary patients (44%). Dignity-related complaints were not significantly associated with MHCA status (χ^2^ = 2.03 and *p* = 0.36). Involuntary patients were more than twice as likely as assisted and voluntary patients to complain about dignity-related matters (Odds ratio [OR]: 2.25; 95% confidence interval [CI] [0.71; 7.13]; *p* = 0.16).

**Conclusion:**

Involuntary patients are more likely to complain about dignity-related matters. Qualitative research is recommended for a deeper understanding of patients’ experiences during admission.

## Introduction

Globally there is a growing interest in promoting the rights of patients, especially psychiatric patients.^1^ Human rights are of particular importance in mental health because, according to law, patients may be admitted and treated involuntarily.^1^ There is also an increasing interest in how psychiatric patients experience mental healthcare services and also in studying this by analysing the complaints lodged by the patients.^2^ Treatment guidelines advocate for a more patient-centred approach.^3^

In South Africa, patients’ rights are ensured by various laws that highlight the importance of treating people in a dignified manner.^4,5,6^ Citizens have the right to take action against the state if their constitutional rights have been infringed.^7,8^ Chapter 2 of the Constitution states that everyone has inherent dignity and the right to have his or her dignity respected and protected.^7,8^ Patients also have the right to complain about the services they receive.^7^ The Patients’ Rights Charter empowers users of health services to contribute towards improving services by giving them a voice to complain.^7,8^

Patients receiving mental healthcare should also be treated in a dignified manner. Dignity is defined as the quality or state of being worthy, honoured or esteemed.^9^ Being treated in a dignified manner may improve patients’ health and help them to cope better with illness.^2,10^ The importance of dignity to healthcare experience appears to be universal. World Health Organization surveys of general medical patients in 41 countries found that being treated in a dignified manner is considered the second most important non-clinical aspect of quality of care, with the most important being the ability to access services promptly.^11^

The Department of Health in South Africa considers patients’ complaints as a valuable resource for monitoring and improving patient safety.^5^ They define a complaint as a dissatisfaction, displeasure, disapproval or discontent expressed about health services being rendered.^5^ To resolve complaints, healthcare institutions usually investigate the complaint and provide feedback to the complainant.^12^

In South Africa, the Office of the Health Ombud is an independent body established by the *National Health Amendment Act* of 2013 to promote accountability.^12^ An ombudsperson was established to represent citizens’ interests and to investigate and deal with complaints concerning public and private sectors.^12^ In the mental healthcare system, the Mental Health Review Board is a second avenue for lodging complaints.^5,13^ Ensuring accountability is especially important with vulnerable populations such as people with mental illness.^12,14^ This was of particular interest in South Africa, during the Esidimeni tragedy, in which 144 psychiatric patients died and 1418 were exposed to torture, trauma and poor health outcomes.^12^ The then minister of health requested that the Health Ombud investigate circumstances around the death of these psychiatric patients and to advise the way forward.^12^

The South African *Mental Health Care Act* 17 of 2002 (abbreviated here as MHCA) has transformed the mental healthcare system to one based on human rights and to redress violations of the past.^5,13^ The MHCA upholds that persons who are mentally ill should receive appropriate care, treatment and rehabilitation, and that their human rights be protected. The MHCA also states that mental healthcare users’ dignity must be respected.^5,6,13^ This, however, remains a challenge to implement in our country, in a healthcare system with limited resources, according to Moosa and Jeenah.^15^

In South Africa, mentally ill patients are admitted to hospital under one of three MHCA-defined legal categories that vary in the level of restriction, namely voluntary, assisted or involuntary.^5,6,13^ The least restrictive and preferred situation is a voluntary admission where the person can consent to admission and treatment.^5,6,13^

Assisted patients agree to treatment, or do not object to treatment, but are incapable of making an informed decision because of their mental illness.^5,6,13^ Involuntary patients are incapable of making an informed decision primarily because of poor insight and impaired judgement and are not willing to receive treatment.^5,6,13^

Concurrently, as a result of mental illness, involuntary patients may be evaluated as likely to inflict harm on themselves, others or property.^5,6,13^

Coercion in mental healthcare is a controversial issue and has been debated throughout history.^16^ Involuntary admission *de facto* entails a restriction of the human rights of psychiatric patients, and they might feel as if their dignity is disregarded.^16,17^ In cases of severe mental illness, patients may be restrained and isolated, which adds to feelings of their dignity not being recognised.^16,17^ Involuntary psychiatric patients are sometimes admitted to hospital for prolonged periods, where they are isolated from their families and communities.^16,17^

A global rise in patient complaints has been accompanied by growing research to analyse complaints for safer, more patient-centric care.^18^ In a systematic review of 59 studies, 88 069 patient complaints were analysed.^18^

The study showed that the most common issues complained about were ‘treatment’ (15.6%) and ‘communication’ (13.7%).^18^ The researchers grouped the complaints into seven categories and then three conceptually distinct domains.^18^ The first domain related to complaints about the safety and quality of clinical care (33.7% of complaints), the second to the management of healthcare organisations (35.1%) and the third to problems in healthcare staff–patient relationships (29.1%).^18^

In South London (UK), a retrospective review was conducted of complaints made by or on behalf of psychiatric patients.^19^ Of 325 recorded complaints, 192 concerned clinical aspects of services.^19^ Poor communication was likely to be at the root of many complaints.^19^ Nearly half of the complaints was that information on their illness and treatment was not given to patients.^19^ This is consistent with other studies conducted in emergency and general healthcare services.^19^ Other common complaints in the South London study were boredom, concerns about privacy, cleanliness, personal safety and safety of possessions.^19^

In another study in mental healthcare services, a complaints register was used to identify and study complaints made in a psychiatric hospital in Northampton (UK).^20^ They analysed 392 complaints, showing that 39% of complaints related to staff behaviour, 26% to clinical matters, 18% to the behaviour of other patients and 16% to the physical environment and facilities.^20^

In this study, we investigated whether there was an association between MHCA status and dignity-related complaints. For this purpose, we studied the complaints lodged to the complaints committee at a specialist state psychiatric hospital. Specific objectives included describing the demographic and clinical profile of patients who lodge complaints, investigating the proportion of dignity-related complaints, and exploring the association between MHCA status and dignity-related complaints.

## Methods

### Study design

We conducted a retrospective, cross-sectional analytical study. Data were obtained from the hospital’s complaints register and clinical files to evaluate the association between MHCA status and dignity-related complaints.

### Setting

The study was conducted at a specialist state psychiatric hospital in South Africa, which is a referral hospital for the neighbouring district hospitals and provides inpatient and outpatient psychiatric services. On admission, patients were assessed and assigned a mental healthcare status under the MHCA, and they are then admitted either in open or in closed wards. Involuntary patients are usually initially admitted in the closed wards, for closer observation and have limited movement as the patients are usually still acutely ill. Often some of these patients may require sedation, isolation and seclusion. Voluntary and assisted patients are usually admitted to the open wards that usually have more lenient rules and where more movement is allowed during admission.

The hospital’s complaints committee is guided by the National Complaints Management Protocol (NCMP) for the Public Health Sector of South Africa.^5^ Established in 2008, the complaints committee consists of hospital staff members, including the chief executive officer, clinical manager, a psychiatrist, deputy director of administration, quality assurance manager, a quality assurance nurse and representatives from social work and psychology. The complaints committee meets weekly to discuss new complaints and review complaints pending investigation and resolutions.

Patients are informed about the complaints system as part of their orientation during admission to the hospital and through regular health education presentations by nursing staff. In each ward, and in the outpatient department, there are posters about how to lodge complaints and boxes in which to post the complaints.

When a patient or a relative has a complaint, the operational ward manager should first assess and see whether the issue can be solved in the ward or at the outpatient department. If the complaint cannot be solved, it is escalated to the complaints committee. The operational manager checks the complaints boxes daily. The complaints are then submitted to the matron’s office and from there to the complaints committee.

Hospital staff members assist complainants who are unable to write and transcribe complaints verbatim. Each complaint is electronically entered into a standardised register linked to the South African Department of Health. For each complaint, the register contains a reference number; date, name and surname of complainant; summary of the events of the complaint; action taken to resolve the complaint; the category of complaints; the severity of the complaint; the type of resolution; the date when the complaint was resolved and the number of days it took to resolve the complaint.

The complaints committee acknowledges receipt of the complaint to the patient within 5 working days, in writing or telephonically. Complaints are assessed immediately for severity or risk, and the appropriate course of action is taken. The complaint is then investigated and a final outcome redressed within 25 working days. Unresolved complaints are referred to the complaints manager or head of the institution. If the complaint cannot be resolved at this level, or the complainant is not satisfied with the resolution, it may be escalated to the District Manager or Provincial Head of Department. This procedure is aligned with the NCMP.^5^

### Participants and procedures

We performed a two-phased sampling process. Firstly, we reviewed 120 complaints lodged in the complaints register over 5 years by inpatients and those which had been escalated to the complaints committee. Complaints were included if the handwriting was legible, the complaint was logical and made sense, and the respective clinical file could be retrieved. Complaints were excluded if the complaints were illegible or seemed to be secondary to a psychotic process, for example, if a patient complained that the treating team was plotting to kill him or her and that the hospital was not a hospital but a prison.

Secondly, we traced and retrieved the complainants’ clinical files from the patient administration department. In this way, we sampled 70 complaints that met the inclusion and exclusion criteria and that were made by 70 different patients. Although a patient might complain about different categories of problems, it would still be handled as a single complaint. For example, a complaint might be that the patient is over-medicated and does not feel safe in the closed ward as other patients are assaulting each other. This complaint would be categorised as a clinical (medication) and a management-related (security) complaint, but it would still be handled as a single complaint. From the clinical files, further details were recorded about demographic characteristics, admission MHCA status and diagnosis.

For each complaint, we categorised the nature of the complaint, that is, whether the complaint pertained to clinical matters, management-related matters or patients’ rights, according to the NCMP.^5^ We recorded the ward the patient was from.

Since dignity has a broad and complex definition, we then defined complaints as dignity-related if they fulfilled any of the following criteria, based on a careful reading of the complaint in the patient’s own words: the complainant felt they were managed in a disrespectful manner, for example, they felt insulted or belittled or the complainant felt they were humiliated or embarrassed, for example, if they were shouted at, assaulted or stripped naked. We also grouped patients’ rights to be falling under dignity. When a complaint was not clear in terms of the category, it was discussed with the research supervisors and a consensus was reached.

To facilitate statistical analysis, we grouped the various *Diagnostic and Statistical Manual of Mental Disorders, Fifth Edition* (*DSM-5*) diagnoses into psychotic disorders, mood disorders, substance-related disorders, personality disorders and cognitive disorders.

### Statistical analysis

Descriptive statistics are presented as proportions. Fisher’s exact tests described the relationship between dignity-related complaints or other categories of complaints and patients’ MHCA status. Logistic regression analyses assessed the association between dignity-related complaints or other categories of complaints, and patients’ MHCA status, adjusted for the potential covariates of staff category (as implicated in the complaint), open or closed wards, patients’ level of education and psychiatric diagnosis.

### Ethical considerations

The chief executive officer of the hospital granted permission to access patient records and to conduct the study at the hospital. The obtained data were kept anonymous and a waiver of written informed consent was given by the Faculty of Health Sciences Research Ethics Committee at the University. Ethics approval for this study was granted by the University of Pretoria Faculty of Health Sciences Research Ethics Committee, Ethics Reference No. 350/2016.

## Results

### Demographic and clinical characteristics

Table 1 describes the demographic characteristics, clinical profile and nature of complaints of the study sample.

**TABLE 1:** Demographic characteristics, clinical profile and nature of complaints (*n* = 70).

Demographic and clinical characteristics	Number	%
**Gender**		
Male	39	56
Female	31	44
**Age (in years)**		
10–19	7	10
20–29	13	19
30–39	22	31
40–49	13	19
50–59	10	14
60+	5	7
**Relationship status**		
Single	43	61
Married	9	13
Divorced	10	14
Widowed	3	4
Separated	4	6
Unknown	1	1
**Highest level of education**		
No education	0	0
Grade 1–7	3	4
Grade 8–11	7	10
Matriculated	16	23
Tertiary education	11	16
Completed tertiary	10	14
Unknown	23	32
**Employment**		
Employed	20	29
Unemployed	34	49
Student	6	9
Pensioner	4	6
Government grant	5	7
Unknown	1	1
**MHCA status**		
Voluntary	18	26
Assisted	10	14
Involuntary	42	60
**Primary psychiatric diagnosis**		
Psychotic disorders	24	34
Mood disorders	39	56
Cognitive disorders	5	7
Personality disorders	1	1
Substance disorders	1	1
**Nature of complaints**		
Categories of complaints	-	-
Clinical	49	70
Management	39	56
Patients’ rights	41	59
Dignity-related complaints	41	58

MHCA, *Mental Health Care Act*.

Most of the complaints were lodged by patients of 30–39 years old (31%) followed by the age group 20–29 years and 40–49 years (both 19%). Most patients (61%) were single, 14% divorced and 13% married. The highest levels of education were mostly matric (23%) and unknown (32%). Patients with mood disorders lodged the most complaints (56%), followed by patients with psychotic disorders (34%). Most of the complainants were admitted involuntarily (*n* = 42; 60%) (Table 1).

### Nature of complaints

Most of the complaints were in the clinical category (70%), followed by patients’ rights (59%) and management (56%) (Table 1). The proportion of clinical complaints was highest amongst involuntary (71%) and assisted patients (70%), followed by voluntary patients (67%) (Table 2). The proportion of management-related complaints was the highest amongst assisted patients (80%), followed by voluntary (61%) and then involuntary patients (48%) (Table 2). The proportion of patients’ rights-related complaints was highest amongst the involuntary patients (62%), followed by assisted (60%) and then voluntary patients (50%) (Table 2).

**TABLE 2:** Association between *Mental Health Care Act* status and clinical, management and patients’ rights complaints.

MHCA status	Number of complaints per category and legal status	Complaints (% per legal status)	Odds ratio	*p*	95% CI
**Clinical**					
Voluntary (*n* = 18)	12	67	1	-	-
Assisted (*n* = 10)	7	70	1.17	0.86	0.21; 6.40
Involuntary (*n* = 42)	30	71	1.25	0.71	0.38; 4.14
**Management**					
Voluntary (*n* = 18)	11	61	1	-	-
Assisted (*n* = 10)	8	80	2.55	0.31	0.38; 16.77
Involuntary (*n* = 42)	20	48	0.58	0.34	0.38; 4.14
**Patients’ rights**					
Voluntary (*n* = 18)	9	50	1	-	-
Assisted (*n* = 10)	6	60	1.5	0.62	0.30; 7.45
Involuntary (*n* = 42)	26	62	1.63	0.4	0.53; 4.95

MHCA, *Mental Health Care Act*; CI, confidence interval.

**FIGURE 1:**
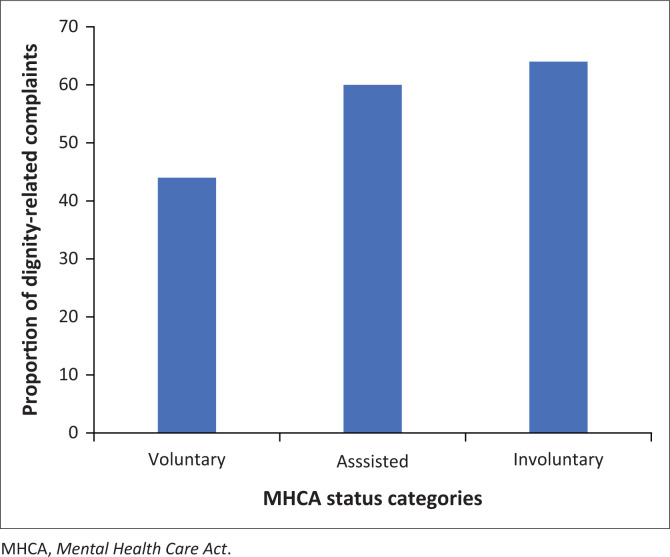
Proportion of dignity-related complaints per *Mental Health Care Act* status category (*n* = 70).

The dignity-related complaints (*n* = 41; 58%) were more than nondignity-related complaints (*n* = 29; 41%) (Table 1). The proportion of dignity-related complaints was higher in involuntary (64%) and assisted (60%) patients than in voluntary patients (44%) (Figure 1; Table 3).

**TABLE 3:** Association between *Mental Health Care Act* status and dignity-related complaints.

MHCA status	Dignity related (*n* = 41)	Dignity related (% per legal status)	Odds ratio	*p*	95% CI
Voluntary (*n* = 18)	8	44	1.0	-	-
Assisted (*n* = 10)	6	60	1.87	0.44	0.37; 9.44
Involuntary (*n* = 42)	27	64	2.25	0.16	0.71; 7.13

MHCA, *Mental Health Care Act*; CI, confidence interval.

### Relationship between patients’ *Mental Health Care Act* status and different categories of complaints

No association was found between clinical complaints and MHCA status (*χ*^2^ = 0.13; *p* = 0.93). In addition, no statistically significant association was observed between management-related complaints and MHCA status (*χ*^2^ = 3.93; *p* = 0.14). Assisted patients were more likely to make management-related complaints (Odds ratio [OR]: 2.55; 95% confidence interval [CI] (0.38; 16.77); *p* = 0.31) (Table 2). No association was found between patients’ rights-related complaints and MHCA status (*χ*^2^ = 0.74; *p* = 0.69). Involuntary patients were more likely to make patients’ rights-related complaints (OR: 1.63; 95% CI [0.53; 4.95]; *p* = 0.40) (Table 2).

### Relationship between patients’ *Mental Health Care Act* status and dignity-related complaints

No statistically significant association was found between dignity-related complaints and MHCA status (*χ*^2^ = 2.03; *p* = 0.36), although involuntary patients were more likely to complain about dignity-related matters (OR: 2.25; 95% CI [0.71; 7.13]; *p* = 0.16) (Table 3).

## Discussion

In this study, complaints were mostly lodged by single, literate male patients aged between 30 and 39 years and diagnosed with a mood disorder. We speculate that this group of patients might have been more likely to be aware of their rights and had the ability to formulate and lodge complaints successfully unlike patients with psychotic disorders.

The issue of the validity of a complaint by a psychotic patient came to the fore in this study. We excluded illogical, illegible complaints or complaints typical of a psychotic process. Patients with psychosis are more likely to hallucinate, have delusions or disorganised thoughts and be unable to formulate valid complaints. For example, Mrs. X who was diagnosed with schizophrenia submitted almost weekly complaints about staff and patients persecuting her and taking her money. She believed that she was the president who was paying their salaries. Not understanding complaints from psychotic patients makes it difficult for committees to assess whether the patients’ human rights are being violated. Conversely, valid complaints may be disregarded on the basis that the complainant may be psychotic, thereby contributing to the violation of their rights.

Although it may generally be assumed that psychotic symptoms are a frequent reason why psychiatric patients complain, our study had only a few complaints that showed a clear psychotic process.^19^ A similarly small contribution from psychosis was found in another study of psychiatric complaints, where only 4% of the complaints was identified as resulting directly from psychotic symptoms.^19^

Our finding that clinical complaints outnumbered complaints in the other categories is in keeping with previous studies where the most common issue complained about was clinical care, followed by management.^18,19^ Another study found that patients often complain that information was not given to them about their illness and treatment.^19^ Engaging with a patient who feels they were admitted against their will and who is refusing treatment can be difficult. Patients might not recall at a later stage that information about their illness and treatment was given to them. Patients may then feel that they were not treated appropriately, and they may subsequently complain.

In our study, it was the involuntary patients who lodged the most complaints. Involuntary patients are admitted and treated without their consent and usually require close observation in closed wards, isolation, continuous monitoring, and sometimes restraint. Research shows that patients treated without their consent often experience humiliating situations that could cause even further psychological and physical trauma.^21,22^

Involuntary procedures may also affect how patients perceive their mental condition as well as treatment outcomes, resulting in patients defaulting treatment, relapses and numerous hospital readmissions.^21,23^ These factors may contribute to more frequent complaints by involuntary patients. Voluntary and assisted patients who might be calmer and willing to be admitted might be admitted in open wards that are usually in a better condition with more lenient ward rules and with more freedom of movement. Patients under voluntary or assisted MHCA status may be less likely to lodge complaints.

Patients who receive involuntary treatment are often considered the most vulnerable and most in need of having their rights protected.^24,25^ Although several laws protect the rights of psychiatric patients and offer avenues for complaints, the priority of treating team is to treat the patient for their mental illness. Providing appropriate treatment in this context is challenging to clinicians who frequently face ethical issues when treating patients without their consent.^26^ It should also be borne in mind that the use of coercion often arises out of complex situations involving clinical, ethical and legal issues.^27,28^

Although our findings are not statistically significant, it is of interest that involuntary patients were more than twice as likely as assisted or voluntary patients to lodge complaints. It is also in keeping with the previous studies which show that patients with severe mental illness may feel that they are being treated in an undignified manner.^27^ The narrower confidence intervals for involuntary patients in comparison with other patients suggest a higher degree of certainty for the estimation of the association between involuntary MHCA status and dignity-related complaints. Advocating for and acting in accordance with the inherent dignity of patients who have severe mental distress, agitation or aggression can be very difficult for a treating team when the team is trying to provide the best evidence-based treatment.^24,26,29^

### Limitations and future research directions

Our findings are based on a small sample from a single specialised institution and may not be generalisable to other settings. The small sample size may be the primary reason for the absence of statistically significant associations. Although not possible at this stage, it would have been helpful to have performed a *post hoc* power calculation based on the variance of the collected data in estimating the possibility of a type II statistical error. This would have provided a more quantified reflection on the strength of our findings.

The researchers also subjectively classified the complaints into categories, one being dignity-related complaints. Dignity is a very broad term with a complex definition. A slightly different classification of complaints might have impacted on the results of this study.

Another limitation of our study is that we did not study the duration of hospitalisation, number of previous admissions as well as whether the patients were admitted in open or closed wards. As previously mentioned, voluntary patients are often admitted in open wards with better conditions and rules than closed wards, usually for a shorter duration than assisted or involuntary patients. This may result in voluntary patients complaining less about dignity-related matters as compared to assisted or involuntary patients.

Our study did not deal with the resolution of complaints, or predictors of complaints, which might be a productive avenue for further research. Another limitation was that specific themes were not explored amongst the dignity-related complaints. Specific dignity-related themes might be better assessed in a qualitative study.

Future qualitative research is needed to develop a deeper understanding of our patients’ experiences and to strengthen therapeutic relationships.

### Clinical recommendations

Our findings suggest that a large proportion of legible, logically phrased complaints are potentially dignity-related, irrespective of the patients’ MHCA status, but even more so for involuntary patients. Ideally, involuntary procedures should be kept to a minimum and only be used when clinically indicated.^27,28^ Healthcare providers should carefully consider the MHCA status of psychiatric patients when deciding on a treatment plan. Involuntary treatment should always be as short as possible and be provided for the sole purpose of restoring competence and good health.^27,28^

We recommend that healthcare providers continue to engage with patients and respect their dignity, even and especially when they are admitted against their will. Shared decision-making is essential in a psychiatric setting.^30^ Healthcare providers must act as advocates for patients’ rights and respect self-determination as far as possible.^4^

Patients and their families should be made aware of their right to complain, and all complaints should be thoroughly investigated. Patients may have pessimistic attitudes because of power hierarchies between staff and patients, especially if they have been involuntarily admitted. Patients admitted involuntarily may also be afraid of lodging complaints.^2^ Given these barriers, all patients should be informed about their rights at the time of admission, especially their right to complain. Psychiatric patients should continuously be informed about their rights whilst they are being treated, as their insight may change during the course of treatment.^31^

Good, clear communication between healthcare providers and patients further helps to establish a good therapeutic alliance, which is vital in the management of patients with mental illness.^21,32^ Good communication also helps mental healthcare providers to assist patients to regain some level of control and a feeling of being treated in a dignified manner.^33^ Patients will feel more respected if there is empathy, involvement and encouragement from healthcare providers.^34^

## Conclusion

Although the results are not statistically significant, our study shows that involuntary patients are more likely than assisted or voluntary patients to lodge complaints about dignity-related matters. In psychiatric settings, it is essential that vulnerable patients, including those admitted and treated without their consent, should be treated in a dignified manner regardless of the challenges. A dignity-related qualitative study is recommended to develop a deeper understanding of the patients’ experiences during admission.
